# Supplementary material for: In Silico Identification of Dysregulated miRNAs Targeting KRAS Gene in Pancreatic Cancer
Source: Diseases. 2024 Jul 12;12(7):152. doi: 10.3390/diseases12070152 (PMC11276408; doi:10.3390/diseases12070152)
Supplement: Supplementary file 1 [file diseases-12-00152-s001.zip › diseases-3073271-supplementary.pdf]

Table S1. miRNAs dysregulated in PC (from dbDMC [23] and "EXP00134" (PMID: 21953293) [24]).

miRID	logFC	AveExpr	*T*	*P*.Value	adj.*P*.Val	B
hsa-miR-199b-5p	−5.80004463462773	7.42237086834135	−14.3493376069262	3.26539468761342E-11	1.74698615787318E-08	15.8165317825896
hsa-miR-216b-5p	−6.30819920466564	6.60464847166827	−10.8329026961837	2.95186511683534E-09	7.89623918753453E-07	11.5364595809623
hsa-miR-216a-5p	−6.56037782149044	6.71265678818865	−9.44449746242995	2.39611909967718E-08	3.06720972969353E-06	9.49144697289844
hsa-miR-145-5p	−3.66049532721094	9.00120750881441	−9.20304685489796	3.52482385675725E-08	3.06720972969353E-06	9.11170356997757
hsa-miR-342-3p	−2.06770871059283	7.06197609054544	−9.17564775404936	3.68423170566382E-08	3.06720972969353E-06	9.06813815214814
hsa-miR-342-5p	−3.1279365158104	2.21423454048632	−9.15533970098284	3.80723604074603E-08	3.06720972969353E-06	9.03578498658273
hsa-miR-210-3p	3.30861759207983	6.6554130067322	9.11798638261719	4.04482253818559E-08	3.06720972969353E-06	8.97613688981463
hsa-miR-34c-5p	4.54366174819511	2.47870583858493	9.03932087836972	4.59728237763139E-08	3.06720972969353E-06	8.84992451071522
hsa-miR-18a-5p	2.95966931229503	4.63122362577916	8.96878272557008	5.15979206864332E-08	3.06720972969353E-06	8.73606301713524
hsa-miR-302c-3p	3.50920517027836	0.988468207342386	8.72322596573032	7.74803868458184E-08	3.90868887134774E-06	8.33455891071302
hsa-miR-425-5p	1.96742546230319	6.32555518879077	8.70135907116401	8.03655655791124E-08	3.90868887134774E-06	8.29841514758857
hsa-miR-100-5p	−2.89826950822513	8.29911197526449	−8.52648241645422	1.0788977753506E-07	4.81008591510475E-06	8.00704512784153
hsa-miR-378a-3p	2.22764330257533	4.42139681596534	8.0912885017575	2.28334892506367E-07	9.39685903776204E-06	7.26383179084238
hsa-miR-892b	3.50499389026838	1.98307478545994	7.8257889356068	3.65106195862589E-07	1.39522724847489E-05	6.7975168368515
hsa-miR-125b-2-3p	−2.93036466161859	2.18986987109058	−7.7319795511438	4.31922754267995E-07	1.54052449022252E-05	6.63038709285579
hsa-miR-18b-5p	2.76126599530068	3.61713524147234	7.61159071260275	5.36803110446388E-07	1.79493540055511E-05	6.41408353188932
hsa-miR-378a-5p	2.3992018863844	1.59811804708316	7.54802112945959	6.02565933030821E-07	1.85832849904423E-05	6.29904004795329
hsa-miR-455-3p	−2.63409516676719	5.85292794838159	−7.52777167962315	6.25232018370021E-07	1.85832849904423E-05	6.26227381188779
hsa-miR-652-3p	1.95529171518872	4.0381557739593	7.41274709899998	7.71948505936387E-07	2.17364447724193E-05	6.05232350075904
hsa-miR-326	1.68572868510695	1.99935341216875	7.37285071769299	8.30848581493981E-07	2.2225199554964E-05	5.97906310954294
hsa-miR-135b-5p	2.38333122828104	6.36931991454141	7.32347532516485	9.10272663875986E-07	2.29467684252872E-05	5.88808349034678
hsa-miR-376c-3p	−3.34959661137798	6.65080761942971	−7.30407385292259	9.43605430572557E-07	2.29467684252872E-05	5.85223928752515
hsa-miR-337-5p	−3.14583592511231	3.34102411068493	−7.12448932061392	1.31950862820121E-06	3.06929180907673E-05	5.51791700573334
hsa-miR-217	−5.1830703871223	5.83660882809215	−6.98173278471156	1.72792862862565E-06	3.85184090131134E-05	5.2488874803506
hsa-miR-148a-3p	−2.92799050399811	11.5063618320805	−6.77642811563132	2.55894631920269E-06	5.47614512309375E-05	4.85692777672182
hsa-miR-15a-5p	1.27801705308878	9.85993882532483	6.51216880613549	4.27786199378564E-06	8.80252371798199E-05	4.34370914069593
hsa-miR-377-3p	−2.38512292472625	5.98634305836432	−6.46733120479629	4.67196197925641E-06	9.25740614408214E-05	4.25566801703862
NA	−2.41232155693022	10.034050531183	−6.32408460698414	6.2023949936286E-06	0.000112072364733802	3.97254950370288
hsa-miR-451a	−3.67536370521478	10.3968744274445	−6.31750120506518	6.28411055360228E-06	0.000112072364733802	3.9594706745804
hsa-miR-26a-5p	−1.46984468668725	11.6094409720137	−6.31747550952679	6.284431667316E-06	0.000112072364733802	3.95941961538528
hsa-miR-564	−2.28347678907003	4.88705702592592	−6.28994106235842	6.63846641516084E-06	0.000113672938799226	3.90465507966451

hsa-miR-200a-5p	2.60234001253119	2.9226038694981	6.2779427507613	6.79912904967334E-06	0.000113672938799226	3.88075908569624
hsa-miR-493-5p	−3.02147147008294	3.03124884710513	−6.22678521508536	7.53056947220428E-06	0.000122086505079675	3.77865596521231
hsa-miR-139-3p	−2.07719309661534	4.43052072625677	−6.20676275530306	7.83856655277483E-06	0.000123342150168663	3.73859858766465
hsa-miR-183-5p	2.42009394597597	3.88113537408191	6.16681622033906	8.49254286142417E-06	0.000126869479750089	3.65852111968284
hsa-miR-144-5p	−3.52590226908824	2.07021557910511	−6.16421639031916	8.53701172150131E-06	0.000126869479750089	3.65330211417875
hsa-miR-144-3p	−4.12331932682993	5.83652929943396	−6.09783775619239	9.7576844535091E-06	0.000141090842773713	3.51974860161836
hsa-miR-455-5p	−1.43261923574542	4.64923908474645	−5.94585580697147	1.32795176278937E-05	0.000186961629761135	3.21179745331671
hsa-miR-499a-5p	−1.61696282465408	1.53090571775788	−5.91068625349751	1.42671783129978E-05	0.000195716420447534	3.14011312651433
hsa-miR-203a-3p	2.29638654925592	4.72815287505182	5.88384780967978	1.50716232376211E-05	0.000201582960803183	3.08530439363481
hsa-miR-149-3p	−2.07371106500371	1.74472397715699	−5.86630700160876	1.56225165709422E-05	0.000203541702742826	3.04943407624386
hsa-miR-182-3p	2.42341125922439	1.46197806291746	5.85529354899452	1.59789747947639E-05	0.000203541702742826	3.02689226816738
hsa-miR-513a-5p	2.21312786458132	1.78626846386932	5.80770845305865	1.7618105024117E-05	0.00021481235068509	2.92932377228532
hsa-miR-194-5p	1.88094048024983	9.78811089858442	5.80636524432752	1.76668101498018E-05	0.00021481235068509	2.92656558491169
hsa-miR-629-3p	2.70270341883933	2.62239837731877	5.78774314540351	1.83565231916588E-05	0.000216399320851893	2.88830347111934
hsa-miR-200c-5p	1.64364400290094	1.39477099269339	5.77819674598934	1.87207887877133E-05	0.000216399320851893	2.868672324356
hsa-miR-196b-5p	4.35964355998847	1.7604293058572	5.77073259577666	1.90107814580168E-05	0.000216399320851893	2.85331532107106
hsa-miR-184	3.86184928090873	0.441236955388609	5.71058888182987	2.15229786521087E-05	0.000239891532893294	2.72932613720817
hsa-miR-572	−2.98972521066757	5.23886275939969	−5.6438283369642	2.47151851949659E-05	0.00026984947100626	2.59118698565737
hsa-miR-337-3p	−2.09252978243534	2.72291645883214	−5.59245787207995	2.75005690716968E-05	0.000294256089067156	2.48453438894103
hsa-miR-338-5p	1.88401665786996	1.38676957887354	5.56671609852545	2.90157717139856E-05	0.000303631695257764	2.43097532739729
hsa-miR-125b-5p	−1.44471504153355	10.8822443012873	−5.55858734315643	2.95118657072966E-05	0.000303631695257764	2.41404655927431
hsa-miR-766-3p	1.97403832298227	4.53452592240287	5.50757953568028	3.28303297250888E-05	0.000326878319336702	2.30764689152057
hsa-miR-576-5p	2.04180678170265	1.84509997143181	5.49954143399755	3.33872318973863E-05	0.000326878319336702	2.29085299295827
hsa-miR-631	2.0683569804329	2.2490609708881	5.49644541217003	3.36043132028385E-05	0.000326878319336702	2.28438258217601
hsa-miR-550a-3p	1.71753986851082	2.35993418462961	5.45572534277467	3.65982030750105E-05	0.000349643547234475	2.19918183297464
hsa-miR-30c-5p	−1.37047913565445	8.37244742361381	−5.40872577767005	4.0396667390162E-05	0.000379161702697135	2.10061519768472
hsa-miR-17-3p	1.59171289988075	4.19284899263953	5.35333839885063	4.53970699824413E-05	0.000418748835182864	1.98415180501055
hsa-miR-501-5p	1.87264420568966	1.72822593235004	5.30011885225109	5.08007166994564E-05	0.000460650566681512	1.87194206702324
hsa-miR-770-5p	1.83171233838558	2.55996362030126	5.22657071209721	5.93738533755396E-05	0.000516732795345379	1.71639383234087
hsa-miR-500a-5p	1.37385969468713	3.15452123062069	5.22140119970184	6.00295708067202E-05	0.000516732795345379	1.70544039400043
hsa-miR-191-5p	1.57161926200469	2.04385602887176	5.22031432753424	6.01683741659009E-05	0.000516732795345379	1.70313713627571
hsa-miR-429	1.66524221545382	8.43274800550985	5.20788120055945	6.17797829258384E-05	0.000516732795345379	1.67678107792204
hsa-miR-1224-3p	2.33586908455371	2.20784906397106	5.20761499266962	6.18147643029986E-05	0.000516732795345379	1.67621659812036
hsa-miR-22-3p	1.23469415801049	10.8287350578727	5.17400213566632	6.63987723931759E-05	0.000546512972774602	1.60488703727234

hsa-miR-27a-3p	1.32979417680422	10.4782958526396	5.13240692718184	7.25568972461838E-05	0.000588150606465277	1.51646864264863
hsa-miR-183-3p	3.04839904200806	1.5884103132414	5.07658157129815	8.17521958187994E-05	0.000648437568027663	1.39754848289431
hsa-miR-650	1.91086298709033	1.28975326991896	5.07279087013565	8.24182329455721E-05	0.000648437568027663	1.38946318836735
hsa-miR-548c-5p	−1.32602485803176	1.61843032612919	−5.02278227910321	9.17426901310874E-05	0.000711338249567127	1.2826792196456
hsa-miR-215-5p	1.65063987212542	9.7925098260741	4.98698575874031	9.90736588198651E-05	0.000757205820980398	1.20610935771368
hsa-miR-381-3p	−1.57429233301942	4.99167171379722	−4.9662772540589	0.000103585219078813	0.000777094500232541	1.16176391146244
hsa-miR-96-5p	1.74658340124309	7.40592119326974	4.96183095449752	0.00010458094208737	0.000777094500232541	1.15223791520299
hsa-miR-495-3p	−2.13484512026306	4.11967387196677	−4.9546101882297	0.000106218861289163	0.000778453298489074	1.13676428904291
hsa-miR-192-3p	1.9127049940124	4.56392607638796	4.90394539693737	0.000118472696893367	0.000851737743888017	1.02807468594176
hsa-miR-106b-5p	1.73952462192934	8.6709388120248	4.89912497985484	0.000119711310052699	0.000851737743888017	1.01772300760966
hsa-miR-93-5p	1.62688657406815	7.36081729788784	4.89418437020523	0.000120994520627083	0.000851737743888017	1.00711134636201
hsa-miR-595	1.8921862275663	2.71974914346143	4.87813320594191	0.000125261121584411	0.000870320779839739	0.972622958779222
hsa-miR-23b-3p	−0.949364078118037	11.011707074214	−4.85554541136313	0.000131526618519344	0.000902137703946781	0.924056464483797
hsa-miR-1228-5p	2.56603555860432	1.74796361811542	4.84707425098694	0.000133957987219594	0.00090718383749978	0.905832605237288
hsa-miR-301a-3p	1.27837764248246	5.29621688061715	4.83530921168087	0.00013741098388816	0.000918611552743217	0.880513909407391
hsa-miR-15b-5p	1.00149272653136	9.33782498288429	4.82973179424884	0.000139079506116263	0.000918611552743217	0.868507618232934
hsa-miR-193a-5p	−1.79103881465606	3.38088644255546	−4.76439629098637	0.000160234297462281	0.00104543108710147	0.727698262985216
hsa-miR-615-3p	1.69284541483973	1.69326527812917	4.75436239921704	0.000163762147990667	0.00105557529126514	0.706047511609592
hsa-miR-222-3p	1.53133263108527	5.17908573071612	4.73081103336751	0.000172356615312923	0.00108561612829583	0.65520317226586
hsa-miR-409-3p	−2.43249501912228	3.44809869707566	−4.73047884889284	0.000172481067112421	0.00108561612829583	0.654485771156327
hsa-miR-512-3p	−1.46685082672327	1.33658715536794	−4.6871809803158	0.000189511095279336	0.00117893530202843	0.560918042607927
hsa-miR-99a-5p	−1.52574205243551	8.60279069550937	−4.67601276136245	0.000194175064447014	0.00119406505148451	0.536764533917112
hsa-miR-329-3p	−1.94445236851294	1.22568715662002	−4.64614089141929	0.000207231956164892	0.00125987609713883	0.47212442096468
hsa-miR-551b-3p	−2.44979150225826	2.75620612675125	−4.55493917301251	0.000252890542906782	0.00151553810383503	0.274467824833883
hsa-miR-376a-3p	−1.30036356082085	7.09943751501829	−4.55122964218875	0.000254950335224584	0.00151553810383503	0.266419346749854
hsa-miR-662	1.40555702771697	0.631706280210988	4.53361895852654	0.000264963815028426	0.00155775429714514	0.228200945101281
hsa-miR-532-3p	−0.934180618324714	4.38019805020013	−4.51749659145525	0.000274480775909492	0.00159616538164759	0.19319985706259
hsa-miR-107	1.02914592589799	9.54054155967665	4.49638051118281	0.000287472292932687	0.00165373845934395	0.147340084798217
hsa-miR-486-5p	−2.62744554084989	3.70598577392053	−4.47527964870056	0.000301077783927379	0.00171358100426753	0.101494401093857
hsa-miR-206	2.60279763308132	1.08838230134172	4.46623757312943	0.000307106093487975	0.00172949221069544	0.081843188960101
hsa-miR-19a-3p	1.20767521015431	8.57811650897741	4.45541425575684	0.000314483080461562	0.00175258800048891	0.0583165537316077
hsa-miR-505-3p	−1.04964888430134	3.55364459770093	−4.45067596744329	0.000317768896475949	0.00175264288262508	0.0480155193520586
hsa-miR-34b-3p	1.46587680877092	3.46854291765604	4.42263386014347	0.000337939860451085	0.00184487576878909	−0.012965010520487
hsa-miR-204-5p	−2.28645652999727	3.66160226809552	−4.39764544233971	0.000357005362690366	0.00192927140443784	−0.0673281344646233

hsa-miR-17-5p	1.42548385184498	6.11457888713375	4.34568017596955	0.000400215111443247	0.00214115084622137	−0.180442552735135
hsa-miR-331-3p	0.858126928540159	7.93654170776586	4.26432353733618	0.000478746092195817	0.0025359322705422	−0.357670690727197
hsa-miR-339-3p	1.13072487771229	2.39291411694157	4.20434270091209	0.0005464653869856	0.00285261695035343	−0.488408233610514
hsa-miR-375	−2.45444218148642	9.54687578709817	−4.20208482640581	0.000549195412871783	0.00285261695035343	−0.493330449956666
hsa-miR-346	1.529601747083	1.2883504142112	4.1926171659195	0.000560793488136687	0.00288485111685699	−0.513970647600397
hsa-miR-640	1.51332556162629	1.21612866217874	4.17194528240793	0.000586982813723107	0.00299081719373202	−0.55903919073373
hsa-miR-145-3p	−1.40168936471401	4.14871212128526	−4.15552886055468	0.000608656766091382	0.00307199405527254	−0.594831692423348
hsa-miR-193a-3p	1.32808938392709	5.40473460525803	4.15057098541031	0.000615359712626358	0.00307679856313179	−0.605641472192544
hsa-miR-141-5p	1.6619719252868	3.37747655947645	4.11567898045923	0.000664684523372733	0.00329265018522604	−0.681718194570034
hsa-miR-99b-3p	1.71004245742267	1.48868586162514	4.10729581504607	0.000677117099905999	0.00332346466467623	−0.699996238314379
hsa-miR-30e-3p	−1.16824741475721	5.56076411123131	−4.08326940287323	0.000714061626511897	0.00347293609258059	−0.752380126419163
hsa-miR-33a-3p	1.89746224789162	0.723021855184875	4.07674268492499	0.00072444341641679	0.00349168673678363	−0.766609511848246
hsa-miR-212-3p	1.29687672280284	4.44327092050331	4.06833678181026	0.000738038422797483	0.00352545139461298	−0.784935376302895
hsa-miR-328-3p	1.2498448638212	3.68844647984663	4.05854728963792	0.000754194966946333	0.00357074608244503	−0.806276856067807
hsa-miR-520b	1.73530558909939	1.15329475497296	4.04063664639659	0.000784681029925889	0.00368249430710834	−0.84532019761985
hsa-miR-543	−2.14228903431711	2.39108013941434	−4.01624747015683	0.000828196422272643	0.00385291379057273	−0.89847946559792
hsa-miR-21-5p	1.14276991962831	13.9504513568501	3.95576226160223	0.000946868046950352	0.00436702073377964	−1.03026907995292
hsa-miR-1236-3p	1.43076970733159	1.76283784870707	3.95096485811643	0.00095698116433699	0.00437593951213923	−1.04071855580362
hsa-miR-638	−2.19949595740882	8.58918144689205	−3.93653377305626	0.000988060405154216	0.00447976539625005	−1.0721480446973
hsa-miR-130a-3p	−0.91122955635773	9.67839544263462	−3.92940181327269	0.00100379179127471	0.00451284544816783	−1.08767864948511
hsa-let-7d-3p	1.3369956644978	2.62757379870457	3.87618444395026	0.00112938182130203	0.00503516061997155	−1.20351508358415
hsa-miR-376b-3p	−1.62858582070189	2.77202349523813	−3.80454930619534	0.00132363602045731	0.00584685154114358	−1.3592705571148
hsa-miR-582-5p	2.51488302818834	4.21982593648522	3.80126571837689	0.00133330072527013	0.00584685154114358	−1.36640449946688
hsa-miR-23b-5p	−1.30555314920261	2.62813711015612	−3.79196263911689	0.00136106751766258	0.00592009042235348	−1.38661352514078
hsa-miR-628-5p	−1.3508509547729	1.75265421423287	−3.76552297173924	0.00144317636515054	0.00622660770448014	−1.44402383737638
hsa-miR-29b-3p	0.958254068099784	10.558073658408	3.75793653507019	0.00146763677292542	0.00628148538812078	−1.46048981664029
hsa-miR-218-5p	−1.31981092468998	6.43186825395912	−3.74368163716287	0.00151472280027926	0.00643156109642385	−1.49142050597252
hsa-miR-363-3p	−2.15623305823038	3.77536260256309	−3.73115579060547	0.00155734027032952	0.00653383980147967	−1.51858958593105
hsa-miR-877-3p	1.4754282787634	3.59571882478367	3.72944981573729	0.00156323643848486	0.00653383980147967	−1.52228918231215
hsa-miR-485-3p	1.3315005965595	2.95825579660629	3.69545033054038	0.00168550036337198	0.0069417478198578	−1.59598246496902
hsa-miR-103a-3p	0.771038157760733	9.41179379783245	3.69510768628478	0.0016867798440776	0.0069417478198578	−1.59672475587237
hsa-miR-140-5p	1.06626791270537	6.96005825005908	3.64260047376369	0.0018947331759685	0.00768955492306851	−1.71037649449549
hsa-miR-30a-3p	−1.31497331760181	6.25494325058941	−3.64200411988281	0.00189723598101877	0.00768955492306851	−1.71166613417342
hsa-miR-609	1.51137721719358	1.34559190448363	3.61857790615791	0.00199819853502469	0.00803786628750531	−1.76230423631423

hsa-miR-1-3p	−3.19235833417753	3.35463788186657	−3.58101989587068	0.00217132867382591	0.00866911074997657	−1.8433955510224
hsa-let-7a-5p	−0.89320419058032	13.3957354765305	−3.56393300372587	0.00225494934339002	0.00893628073121229	−1.88024714470095
hsa-miR-483-3p	1.36751747678743	3.46903477485818	3.55595769384781	0.00229506624888192	0.00902838561141048	−1.89743853814664
hsa-miR-199a-5p	−1.19275202001213	9.5361906019014	−3.54350396550061	0.00235913137936977	0.00921266633549511	−1.92427162527583
hsa-miR-181c-3p	1.25125266280605	1.87933344188242	3.5125332397653	0.00252624747481607	0.00979378550019272	−1.99093681257447
hsa-miR-152-3p	0.987642920670542	5.40908164756492	3.50710494813052	0.00255672397388114	0.00981659442318177	−2.00261146193649
hsa-miR-140-3p	0.873515767518865	7.04830925249194	3.50496674500391	0.00256882844718775	0.00981659442318177	−2.00720927550859
hsa-miR-371a-5p	1.79975533370617	2.23364186381064	3.49941613277391	0.00260051643139256	0.00984426015726218	−2.01914264411684
hsa-miR-192-5p	1.40901166527392	10.4535692419838	3.49727052560044	0.00261286905108641	0.00984426015726218	−2.02375466802949
hsa-miR-1229-3p	1.01107496323828	2.40291205366942	3.4901443819646	0.00265431407669218	0.0099210724425499	−2.03906898351427
hsa-miR-126-5p	−1.08607227423074	3.67715222181945	−3.4874175581847	0.00267034473220035	0.0099210724425499	−2.04492759617664
hsa-miR-188-5p	1.72784638581523	5.0605984578609	3.4650882381911	0.00280527421280953	0.0103504945093317	−2.09287221176298
hsa-miR-513b-5p	1.47233811594416	1.23407580218056	3.44346025528997	0.00294239211052006	0.0107820532817002	−2.1392581479148
hsa-miR-885-5p	1.41456531522158	4.09842715157828	3.42848265721214	0.00304121156417673	0.0109976929683275	−2.17134933214661
hsa-miR-141-3p	1.33832764048804	11.4808818371983	3.42831256338621	0.00304235244731304	0.0109976929683275	−2.17171362573706
hsa-miR-214-3p	−1.16634037181386	8.4817405077237	−3.42435744326712	0.00306900052995916	0.0110195656612627	−2.18018342098656
hsa-miR-501-3p	−0.932089623829353	2.50048932243768	−3.41382188797898	0.00314111590841541	0.0112033134066816	−2.20273590476028
hsa-miR-939-5p	−1.46555746343283	7.11149935093272	−3.384974807116	0.00334725046379273	0.0118594635637689	−2.2644163137725
hsa-miR-200a-3p	0.983028497294892	9.97519819049299	3.36100546023915	0.00352864196229739	0.0123327259081444	−2.31558655250084
hsa-let-7b-5p	−0.820444704957138	12.4927623400727	−3.35880198527041	0.00354579580905073	0.0123327259081444	−2.32028678688877
hsa-miR-197-3p	1.406708781118	6.1251612565651	3.35673665911653	0.00356194882519084	0.0123327259081444	−2.32469174792536
hsa-miR-654-3p	−1.58836589973048	4.53185386247168	−3.35532480442382	0.00357303273974277	0.0123327259081444	−2.32770264604009
hsa-miR-520d-3p	1.19180647521281	1.05923623898951	3.33452523295872	0.00374031661156792	0.0128273678665951	−2.37202815915554
hsa-miR-99a-3p	−1.97042239503146	1.11824338335736	−3.31181325350906	0.00393180005567087	0.0133981721642288	−2.42036057053325
hsa-miR-299-3p	−1.52226550858336	2.06895201638628	−3.29451447235463	0.00408408930788206	0.013770456914655	−2.45712379608185
hsa-miR-379-5p	1.27934477762961	4.72455467116825	3.29357452556288	0.00409252831669186	0.013770456914655	−2.45912010901426
hsa-miR-491-3p	1.42601290266983	3.52659370528142	3.28932167797349	0.00413092601105776	0.0138127838494744	−2.46815091666154
hsa-miR-489-3p	−1.00161969607516	0.938609254167242	−3.27988017368054	0.00421744144724639	0.0140144793433343	−2.48819005961032
hsa-miR-26b-5p	−0.6559209536995	10.9202142713446	−3.17007291700952	0.00536406054452957	0.0177146443908847	−2.72021314918796
hsa-miR-199a-3p	−1.25921483625932	11.5894366405258	−3.15997396882754	0.00548371080917491	0.0179181421192095	−2.74145024368267
hsa-miR-610	1.32620630608312	0.989263321636346	3.1592269691101	0.00549266412626236	0.0179181421192095	−2.74302038955103
hsa-miR-30b-5p	−0.784513135015754	9.52521306233389	−3.13424374074298	0.00580048215585556	0.0188076239598953	−2.79547541203098
hsa-miR-16-5p	0.613101965935586	11.6053083176554	3.12352547143717	0.00593765367335915	0.0190629932134803	−2.81794442301769
NA	−1.45950063908586	5.14979413535512	−3.12253376994176	0.00595050442364711	0.0190629932134803	−2.82002227033444

hsa-miR-196a-5p	2.81402377467692	2.01497815713802	3.11295257813742	0.00607606774441775	0.0193493824003779	−2.84008756631329
hsa-miR-150-5p	−1.36971173200861	6.09367859414417	−3.08984481447238	0.00638963791345947	0.020227551974561	−2.88840847637712
hsa-miR-654-5p	−1.07807011266218	2.31900715545942	−3.08468754726145	0.00646174757332219	0.0203354997160434	−2.89917872756343
hsa-miR-937-3p	1.28237894509856	2.33173993533392	3.07993678942846	0.00652887530881472	0.0204265981883969	−2.90909539931984
hsa-miR-28-3p	−1.11077322412118	3.0877935047843	−3.03670027731229	0.00717187832233255	0.0223078773398135	−2.99913761560248
hsa-miR-548d-3p	−1.69814321072137	0.608535963729215	−2.97380055680865	0.00821855351675745	0.0254157579853482	−3.12942513513065
hsa-miR-211-5p	1.01190825728933	1.23620113588337	2.96598105032569	0.00835861040080089	0.0257003250829223	−3.14556103919973
hsa-miR-300	1.5561234716663	1.23226990829216	2.94853645176642	0.00867945690400516	0.0264913184861982	−3.18150839736055
hsa-miR-29a-3p	0.716119142805978	11.1742203818105	2.94664782196785	0.00871490103471193	0.0264913184861982	−3.18539600233617
hsa-miR-647	1.48942247076966	2.36836981716988	2.93632805766582	0.00891106377677891	0.0269345713026933	−3.20662376272502
hsa-miR-136-3p	−1.34226388495609	4.22941931492129	−2.9158978933101	0.0093120983970725	0.0279886103507516	−3.24857434416269
hsa-miR-622	−0.874610171372925	1.99314024191419	−2.87681410868132	0.0101284833492114	0.0302530703348909	−3.32854529146704
hsa-miR-202-3p	0.976593564864035	2.96072249746362	2.87451494032674	0.0101786031033278	0.0302530703348909	−3.33323789965901
hsa-miR-93-3p	1.18120535776923	0.974233668823673	2.84724816456594	0.0107915478051696	0.0316878312270964	−3.38878680997333
hsa-miR-29c-5p	−1.11644148262407	5.189680458659	−2.84443422857477	0.0108567931474244	0.0316878312270964	−3.39450856315022
hsa-miR-32-3p	1.27522500744673	3.35233647697829	2.84436966400399	0.0108582946274908	0.0316878312270964	−3.39463982215531
hsa-miR-181a-2-3p	−1.06028673859067	2.35501244203909	−2.842654936673	0.010898244758478	0.0316878312270964	−3.39812544449577
hsa-miR-30c-2-3p	−0.957460139877897	3.23346560642011	−2.82579897491899	0.0112985729728961	0.032674251570267	−3.43234840228247
hsa-miR-20a-5p	0.701690642846335	9.69725133712984	2.80142824260019	0.0119025008277837	0.0342356878648619	−3.48169479141015
hsa-miR-431-5p	1.43425222369111	1.10398442396316	2.77500097301473	0.0125924475376056	0.0358620390881486	−3.53502205373168
hsa-miR-10b-5p	0.857774339942811	7.54322603782826	2.77464539774407	0.0126019875674242	0.0358620390881486	−3.53573824159053
hsa-miR-760	0.848671317403599	1.61632192315417	2.75853234037113	0.0130416536993846	0.0369168504188928	−3.56815521257343
hsa-miR-139-5p	−0.915624500327887	3.87421283133097	−2.75429315458542	0.013159748498382	0.0370550812980755	−3.57667154674322
hsa-miR-605-5p	1.10860801683657	2.23502360004694	2.73353694055008	0.0137529264498544	0.0385225950296968	−3.61829515829768
hsa-miR-194-3p	1.25815380528072	2.83457907546969	2.70780008336736	0.0145240530239426	0.04047066858234	−3.66973151427199
hsa-miR-27b-3p	−0.537123260233383	11.0777687572947	−2.70514279400373	0.0146059860435608	0.0404880960274871	−3.67503100580937
hsa-miR-143-5p	−1.15942977783555	2.61254257257968	−2.69672431634216	0.0148684832389378	0.041003291406349	−3.69180611733386
hsa-miR-361-5p	−0.638114948187379	7.18031975742313	−2.68529386331375	0.0152321215770498	0.0416953673618337	−3.71454862152299
hsa-miR-182-5p	1.36363334053817	4.26356805058936	2.68395358794497	0.0152753121549895	0.0416953673618337	−3.717212673557
hsa-miR-30e-5p	−0.68454941871983	8.60919645540383	−2.66368480272513	0.0159429008390386	0.0432967104004348	−3.75743308847655
hsa-miR-877-5p	1.71076394401906	1.01769044068072	2.64998286593951	0.0164098661041619	0.044339789725892	−3.78454984860182
hsa-miR-362-3p	−0.739659077510305	4.6741998180404	−2.64114386999799	0.0167179863927064	0.0449453403019997	−3.80201107420821
hsa-miR-563	−0.810820650725805	1.92179816854758	−2.62982045236865	0.017120766941216	0.0457980515677527	−3.82434367050411
hsa-miR-133b	−1.84824348347107	3.10581590417739	−2.62468472225625	0.0173064767930183	0.0459018241913836	−3.83445896896066

hsa-miR-30a-5p	−0.824184728184234	9.75104474609562	−2.6240061780748	0.0173311560498308	0.0459018241913836	−3.83579478488711
hsa-miR-135a-3p	1.58391400771188	3.4306150203481	2.62024789654295	0.0174684554196563	0.0460375549237247	−3.84319081231271
hsa-miR-454-5p	0.904199882059901	1.79305950827011	2.6101768377175	0.0178414939498706	0.0467901924665723	−3.86298716700224
hsa-miR-202-5p	−0.977833612678688	0.28856682032084	−2.60758838031086	0.0179385882543052	0.0468153400783087	−3.86806983075293
hsa-miR-518a-3p	−1.93812668822714	0.630335660887334	−2.601952283533	0.0181517406025419	0.0471416564192229	−3.87912914392035
hsa-miR-181d-5p	0.757449405757753	4.71478743329594	2.58900075979331	0.0186507074727509	0.0481336041120413	−3.90450296572814
hsa-miR-33a-5p	0.948079357287269	3.39617412755333	2.58739102838973	0.0187136255239338	0.0481336041120413	−3.90765272925706
hsa-miR-29c-3p	−0.746819542159868	11.5103107354528	−2.57774438010549	0.01909491492306	0.0488793276738618	−3.92651003244449
hsa-miR-376a-5p	−1.16877542538614	1.19487335208702	−2.5666399731638	0.019542942737811	0.0497879731653757	−3.9481778546508

miRID: Identifier for the miRNA; **logFC:** Log Fold Change; **AveExpr:** Average expression level of the miRNA; **t:** t-value from the t-test; ***P*:** *P* value; **adj.*P*.Val:** Adjusted *P*-value; **B**: B-statistic.

Table S2. miRNAs dysregulated in PC and targeting *KRAS* (from dbDMC [23] and "EXP00134" (PMID: 21953293) [24]).

miRID	Cancer Type	Design	logFC	AveExpr	*T*	*P* value	Adj.*P*.Value	Status
hsa-miR-885-5p	pancreatic cancer	cancer vs normal	1.41	4.1	3.43	3.04E-03	1.10E-02	UP
hsa-miR-877	pancreatic cancer	cancer vs normal	1.71	1.02	2.65	1.64E-02	4.43E-02	UP
hsa-miR-654-3p	pancreatic cancer	cancer vs normal	−1.59	4.53	−3.36	3.57E-03	1.23E-02	DOWN
hsa-miR-647	pancreatic cancer	cancer vs normal	1.49	2.37	2.94	8.91E-03	2.69E-02	UP
hsa-miR-548c-5p	pancreatic cancer	cancer vs normal	−1.33	1.62	−5.02	9.17E-05	7.11E-04	DOWN
hsa-miR-513b	pancreatic cancer	cancer vs normal	1.47	1.23	3.44	2.94E-03	1.08E-02	UP
hsa-miR-495	pancreatic cancer	cancer vs normal	−2.13	4.12	−4.95	1.06E-04	7.78E-04	DOWN
hsa-miR-377	pancreatic cancer	cancer vs normal	−2.39	5.99	−6.47	4.67E-06	9.26E-05	DOWN
hsa-miR-34b	pancreatic cancer	cancer vs normal	1.47	3.47	4.42	3.38E-04	1.84E-03	UP
hsa-miR-326	pancreatic cancer	cancer vs normal	1.69	2	7.37	8.31E-07	2.22E-05	UP
hsa-miR-30e	pancreatic cancer	cancer vs normal	−0.68	8.61	−2.66	1.59E-02	4.33E-02	DOWN
hsa-miR-30b	pancreatic cancer	cancer vs normal	−0.78	9.53	−3.13	5.80E-03	1.88E-02	DOWN

hsa-miR-30a	pancreatic cancer	cancer vs normal	−0.82	9.75	−2.62	1.73E-02	4.59E-02	DOWN
hsa-miR-222	pancreatic cancer	cancer vs normal	1.53	5.18	4.73	1.72E-04	1.09E-03	UP
hsa-miR-217	pancreatic cancer	cancer vs normal	−5.18	5.84	−6.98	1.73E-06	3.85E-05	DOWN
hsa-miR-181d	pancreatic cancer	cancer vs normal	0.76	4.71	2.59	1.87E-02	4.81E-02	UP

miRID: Identifier for the miRNA; **logFC:** Log Fold Change; **AveExpr:** Average expression level of the miRNA; **t:** t-value from the t-test; ***P*:** *P* value; **adj.*P*.Val:** Adjusted *P*-value; **B**: B-statistic; **DOWN**: downregulated; **UP:** upregulated.

Table S3. Association of miRNAs and clinical features of PC (from dbDMC [23] and "EXP00134" (PMID: 21953293) [24])**.**

miRNA Name	Clinical Parameter	ANOVA P-value	ANOVA FDR	Multivariate Log Rank P-value	Multivariate Log Rank FDR
hsa-miR-222-3p	Histologic Grade	0.0175	0.0556	0.402	0.147
hsa-miR-222-3p	Pathologic N Status	0.191	0.556	0.300	0.0369
hsa-miR-30a-5p	Pathologic M Status	0.421	0.823	0.118	0.0192
hsa-miR-30a-5p	Pathologic Stage	0.0143	0.0714	0.200	0.034
hsa-miR-30a-5p	Pathologic T Status	0.269	0.533	0.247	0.039
hsa-miR-30a-5p	Sex	0.395	0.856	0.0981	0.0168
hsa-miR-30b-5p	Histologic Grade	0.158	0.290	0.224	0.0446
hsa-miR-30b-5p	Pathologic M Status	0.00669	0.165	0.226	0.0704
hsa-miR-30e-5p	Pathologic N Status	0.946	0.974	0.312	0.0405
hsa-miR-30e-5p	Pathologic T Status	0.828	0.999	0.270	0.0491
hsa-miR-377-3p	Pathologic Stage	0.00287	0.0233	0.343	0.122
hsa-miR-377-3p	Pathologic T Status	0.000632	0.00621	0.402	0.146
hsa-miR-495-3p	Histologic Grade	0.0251	0.0724	0.195	0.0343
hsa-miR-495-3p	Pathologic M Status	0.0204	0.259	0.195	0.0474
hsa-miR-495-3p	Pathologic N Status	0.0404	0.258	0.315	0.0437
hsa-miR-495-3p	Pathologic Stage	0.0129	0.0671	0.222	0.0468
hsa-miR-495-3p	Pathologic T Status	0.00153	0.0119	0.263	0.0443
hsa-miR-495-3p	Sex	0.905	0.984	0.154	0.0364
hsa-miR-654-3p	Histologic Grade	0.0177	0.0556	0.751	0.469
hsa-miR-654-3p	Pathologic T Status	0.0394	0.13	0.635	0.344
hsa-miR-877-5p	Pathologic T Status	7.97e-05	0.00139	0.645	0.353
hsa-miR-885-5p	Histologic Grade	1.73e-05	0.000302	0.267	0.0673

hsa-miR-885-5p	Pathologic N Status	0.00522	0.0706	0.381	0.0783
hsa-miR-885-5p	Pathologic Stage	0.0268	0.117	0.302	0.0876
hsa-miR-885-5p	Pathologic T Status	2.11e-09	2.33e-07	0.329	0.0856
hsa-miR-885-5p	Sex	0.0675	0.854	0.175	0.0496
